# Genetic diversity and population structure of *Caryopteris mongholica* revealed by reduced representation sequencing

**DOI:** 10.1186/s12870-022-03681-y

**Published:** 2022-06-17

**Authors:** Ruoxuan Ji, Xiao Yu, Tianmeng Ren, Yuan Chang, Zheng Li, Xinli Xia, Weilun Yin, Chao Liu

**Affiliations:** College of Biological Sciences and Biotechnology, National Engineering Research Center of Tree Breeding, Beijing Forestry University, Beijing, China

**Keywords:** *Caryopteris mongholica*, Genetic diversity, Population structure, Genotyping by sequencing, Environmental

## Abstract

**Background:**

*Caryopteris mongholica* Bunge is a rare broad-leaved shrub distributed in the desert and arid regions of Mongol and North China. Due to land reclamation, natural habitat deterioration and anthropogenic activities in recent years, the wild resources have sharply reduced. To effectively protect and rationally use it, we investigated the genetic diversity and population structure from 18 populations across the range of *C. mongholica* in China by reduced representation sequencing technology.

**Results:**

We found the overall average values of observed heterozygosity (*Ho*), expected heterozygosity (*He*), and average nucleotide diversity (*π*) were 0.43, 0.35 and 0.135, respectively. Furthermore, the NM17 population exhibited higher genetic diversity than other populations. The phylogenetic tree, principal component analysis (PCA) and structure analysis showed the sampled individuals clustered into two main groups. The NM03 population, with individuals clustered in both groups, may be a transitional population located between the two groups. In addition, most genetic variation existed within populations (90.97%) compared to that among the populations (9.03%). Interestingly, geographic and environmental distances were almost equally important to the observed genetic differences. Redundancy analysis (RDA) identified optical radiation (OR), minimum temperature (MIT) and mean annual precipitation (MAP) related variables as the most important environment factors influencing genetic variation, and the importance of MIT was also confirmed in the latent factor mixed models (LFMM).

**Conclusions:**

The results of this study facilitate research on the genetic diversity of *C. mongholica*. These genetic features provided vital information for conserving and sustainably developing the *C. mongholica* genetic resources.

**Supplementary Information:**

The online version contains supplementary material available at 10.1186/s12870-022-03681-y.

## Background

*Caryopteris mongholica* Bunge (*Caryopteris*, Lamiaceae) is an endangered shrub mainly distributed in the arid and semi-arid areas of Mongolia and North China (Gansu, Hebei, Inner Mongolia, Shanxi provinces etc. [1]). According to our long-term field investigations, violet-blue was the most common flower color of *C. mongholica* and it is rarely pink. Additionally, *C. mongholica* had mixed mating system with both inbreeding and outcrossing [2]. As the northernmost species within the genus *Caryopteris* [3], *C. mongholica* has an important phylogenetic position. Climate-induced local extinction is widespread among plant and animal species [4, 5]. Recent land reclamation, natural habitat deterioration and anthropogenic activities have sharply reduced the wild resources *C. mongholica* [6, 7]. Due to the important roles that *C. mongholica* plays in fixing moving sands, water and soil conservation, and delaying further desertification for the ecological-environmental stability of native habitats, the protection and research are becoming particularly important and necessary.

*C. mongholica* has high morphological and genetic diversity, owing to its wide geographic distribution and the survival in a broad range of environmental conditions [7, 8]. For example, the population density of *C. mongholica* in Saihan Tal, Inner Mongolia, is less than 20% compared with that in Shenmu, Shaanxi Province, China, where it is more than 40%. A phenotypic study of six *C. mongholica* populations from different areas showed that variable climatic factors and environmental conditions contributed to high trait variation, modulating photosynthetic responses and growth [8, 9]. Under the drought conditions, the growth and photosynthetic capacity of *C. mongholica* are inhibited, and the moisture capacity of leaves decreases [9]. Wu [7] first characterized *C. mongholica* germplasm with inter-simple sequence repeat markers and confirmed a high level of a genetic variation among five *C. mongholica* populations of in northwestern China, while genetic differentiation within populations was higher than that among populations, and clustering analysis showed populations groups geographically structured. To a certain extent, the heterogeneous environments across the geographic range of *C. mongholica* induced the differences in the community structure, plant morphology and genetic structure, which diversified of *C. mongholica*.

Single nucleotide polymorphisms (SNPs) are the most abundant and universal sequence variations in all genomes, which makes them very useful markers for genetic analysis [10]. With the rapid development of high-throughput sequencing technology, so far, scientists have developed various reduced-representation genome sequencing by restriction enzyme digestion of genomic DNA [11, 12]. Genotyping by sequencing (GBS) method is increasingly applied in numerous species [13, 14], which is a high-performance, cost-effective, and simple, allowing obtaining thousands of markers from many individual and identifying SNPs using a reduced representation library [11, 13, 15]. Furthermore, GBS detects results in SNP markers that are more informative than PCR-based markers [16, 17].

Early study relating to *C. mongholica* genetic diversity has relied on traditional molecular marker (inter-simple sequence repeat). However, this marker has its own limitations in resolving the geographical differentiation of population, especially when using few genetic markers. Here, we identified SNPs of *C. mongholica* from 18 natural populations in Northwest China using GBS to address the above drawbacks. We aimed to answer the following questions with the detected SNPs: (1) what is the level of genetic diversity and population structure of *C. mongholica* in these different populations? (2) does geographic and environmental distance affect the genetic diversity of *C. mongholica*? (3) what is the relative importance of environmental variables to the genetic diversity of *C. mongholica*? According to the results, at the last, we provided a reference for the protection and rational utilization of the wild *C. mongholica* resources.

## Results

### SNPs discovery, genotype and population genetic diversity

Our sequencing data showed high phred quality (Q20 > 94%, Q30 > 85%), with a stable GC content ranging from 34.38% to 38.33%. Sequencing generated 126 billion paired-end reads from 125 individuals, of which 99.99% reads passed initial quality filters (Additional file 1: Table S1 and S2). Under our parameter settings, SAM tools initially recovered 165,622 SNPs. There were 9,763 SNPs after filtering based on missing data, allele frequency, and depth that were used for all analyses. With all 125 individuals, as a whole, the *Ho*, *He*, and *π* ranged from 0.35(NM14) to 0.73(NM17), 0.28(NM14) to 0.70(NM17), 0.126(NX07) to 0.151(NM17), respectively, with the means of 0.43, 0.35, and 0.135, respectively (Table 1).

**Table 1:** Geographic locations and genetic diversity in 18 populations of *C. mongholica*

Population	Longitude (° E)	Latitude (° N)	Altitude (m)	Cluster	*Ho*	*He*	*π*	*F*_*IS*_	*Nm*
NM01	111.22	40.77	1 123	B	0.40	0.32	0.139	-0.070	−
NM02	109.58	39.94	1 357	B	0.39	0.32	0.132	-0.057	−
NM03	108.04	39.25	1 396	B	0.37	0.31	0.134	-0.056	−
NX06	105.14	37.66	1 193	A	0.45	0.37	0.127	-0.053	−
NX07	105.98	38.69	1 329	A	0.47	0.39	0.126	-0.044	−
NM08	107.61	38.41	1 326	A	0.40	0.32	0.141	-0.069	−
NM09	108.55	37.72	1 321	B	0.40	0.32	0.138	-0.067	−
SX10	109.71	38.33	1 090	B	0.39	0.32	0.137	-0.064	−
SX11	110.33	39.29	1 206	B	0.40	0.32	0.136	-0.068	−
NM13	111.68	39.65	1 143	B	0.39	0.31	0.137	-0.063	−
NM14	111.93	40.41	1 299	B	0.35	0.28	0.129	-0.074	−
NM16	111.77	41.10	1 625	B	0.40	0.32	0.135	-0.065	−
NM17	112.43	42.57	1 269	B	0.73	0.70	0.151	-0.023	−
NM18	112.02	43.58	971	B	0.40	0.32	0.137	-0.066	−
NM19	115.35	43.90	1 017	B	0.49	0.39	0.139	-0.065	−
NM20	116.76	45.66	1 038	B	0.40	0.32	0.131	-0.059	−
HB23	114.85	41.99	1 590	B	0.41	0.33	0.135	-0.064	−
GS01	103.74	36.10	1624	A	0.43	0.33	0.127	-0.074	−
Mean					0.43	0.35	0.135		−
Species level									3.002

*Ho* Observed heterozygosity, *He* Expected heterozygosity, *π* Average nucleotide diversity, *F*_*IS*_ Inbreeding coefficient, *Nm* Gene flow

### Phylogenetic relationship

**Fig. 1:**
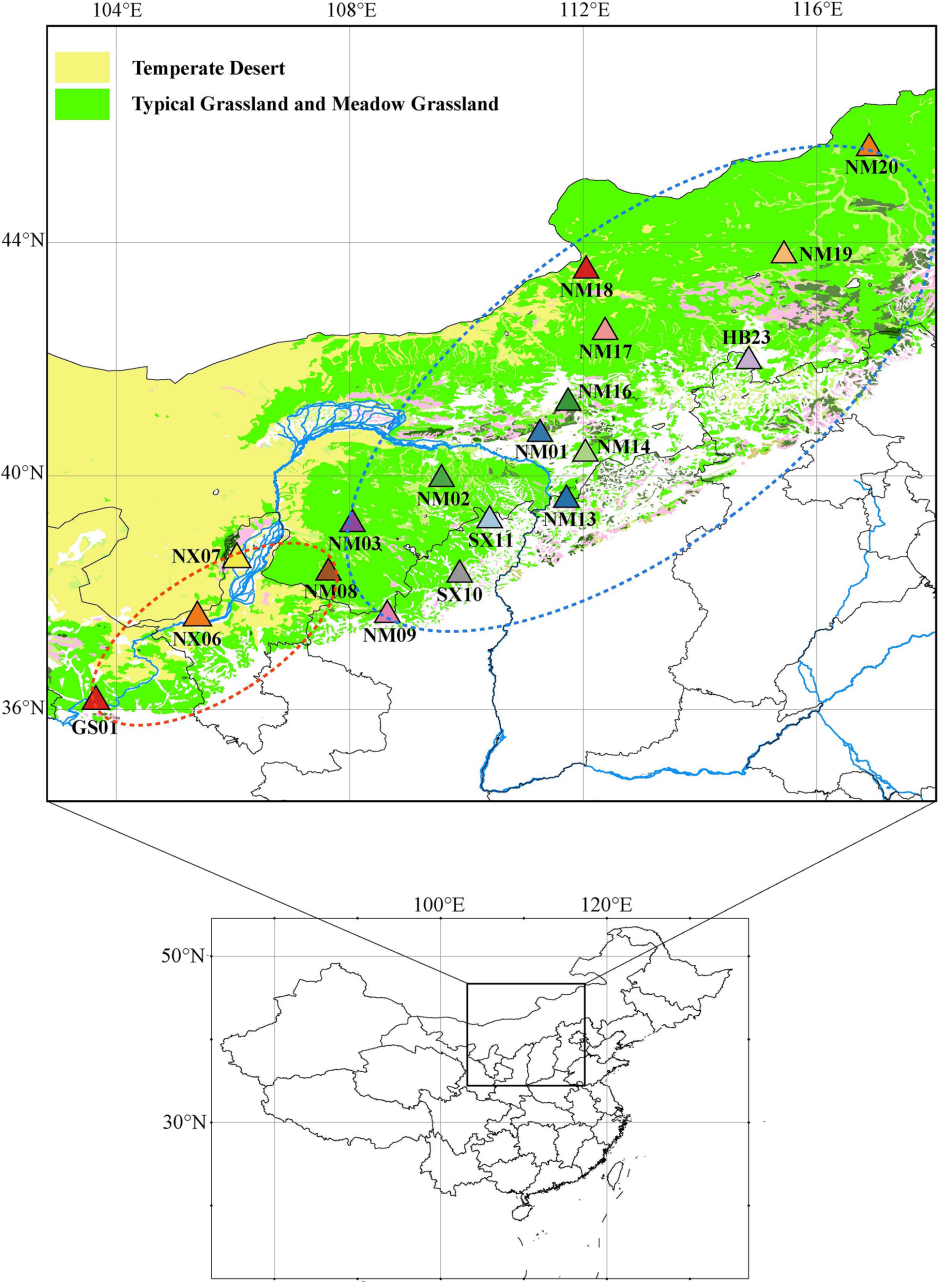
Location of the 18 natural sample sites of China collection

**Fig. 2:**
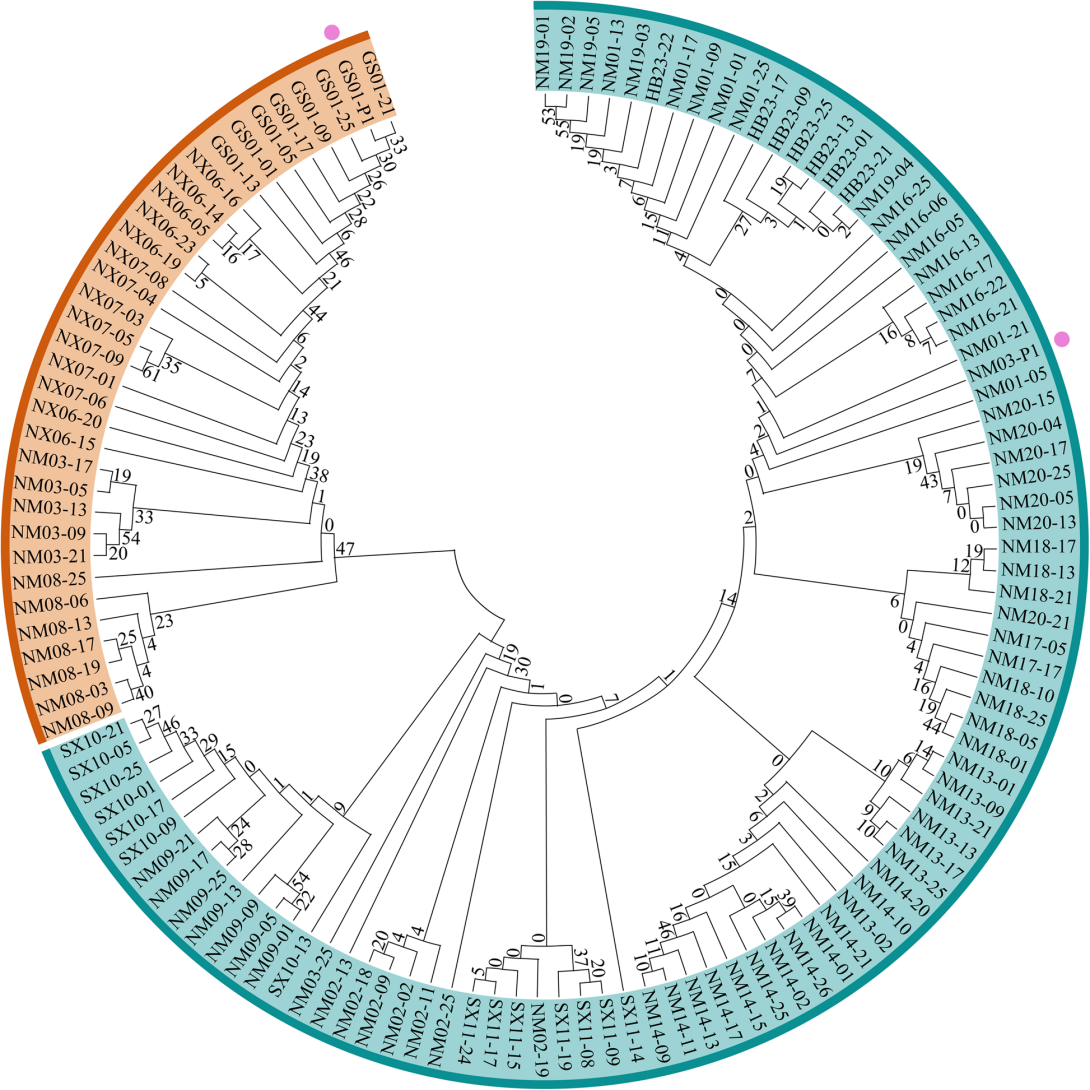
Phylogenetic analysis of *C. mongholica*. Circular maximum likelihood tree based on SNPs showing the phylogenetic relationship of the 125 individuals. The color pattern are equivalent to the structure analysis at *K* = 2 (orange = group A, green = group B). The flower of the marked (pink dots) individual was pink. Numbers at the nodes are bootstrap values from 100 replications

To better visualize sample distribution and the relationships, a phylogenetic trees of 125 *C. mongholica* individuals were constructed (Fig. 2). Within the population, the individuals of each population get together except NM01, NM02, NM03, SX10, and NM20, but there are clear genetic boundaries between different populations. Four populations (NX06, NX07, NM08 and GS01) and five individuals of NM03 were clustered in a small group, which was distributed on the southwest side of the sampling locations around Helan Mountain (~ 103 – 107ºE, ~ 36 – 38ºN) (Fig. 1). While the other 13 populations and the remaining two individuals of NM03 were clustered in a large group, which was on the northeast side of the sampling locations in the Daqing Mountain and its surrounding grasslands (~ 108 – 116ºE, ~ 38 – 45ºN) (Fig. 1). Visibly, most genetic variation occurs primarily within populations of *C. mongholica*. For color of flowers, the “NM03-P1” was clustered in the Daqing Mountain-Grassland group, gathered on the branch with NM16 population, and “GS01-P1” was clustered in the Helan Mountain group, gathered on the branch with GS01 population (Fig. 2).

Some populations of *C. mongholica* clustered in the same group were geographically distant, such as NM02 and NM20, whereas NM08, which was more closer to NM02, clustered in different groups (Fig. 1 and Fig. 2).

### Population analyses of genotyping results

**Fig. 3:**
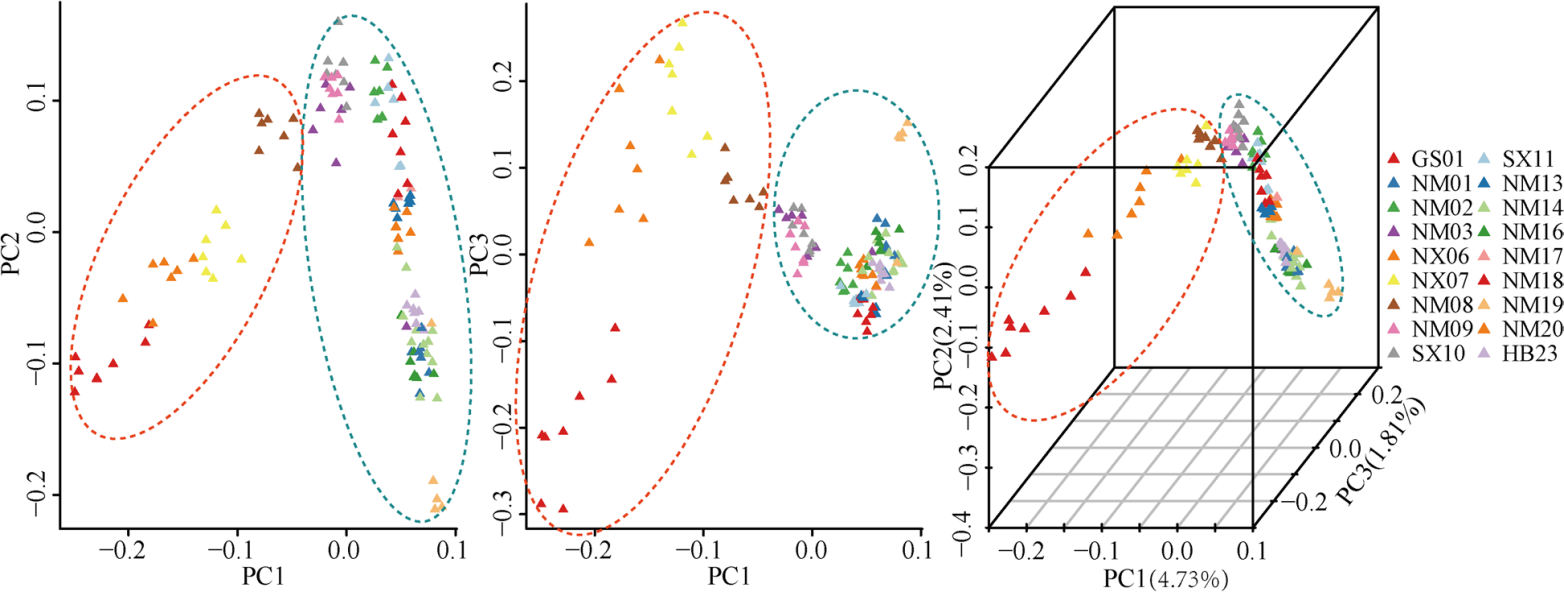
Principal component analysis (PCA)-based clustering of the 125 *C. mongholica* individuals (orange circle = group A, blue circle = group B)

As expected, analysis of unlinked SNPs for 125 individuals performed with the principal component analysis (PCA) showed two groups corresponding to the 18 populations of *C. mongholica* (Fig. 3). The Helan Mountain group (orange circle) included four populations (NX06, NX07, NM08 and GS01), and the Daqing Mountain-Grassland group (blue circle) included all the remaining 14 populations (NM01, NM02, NM03, NM09, SX10, SX11, NM13, NM14, NM16, NM17, NM18, NM19, NM20 and HB23). Geographically, these two genetic groups corresponded to the southwest and northeast populations, respectively (Fig. 3).

**Fig. 4:**
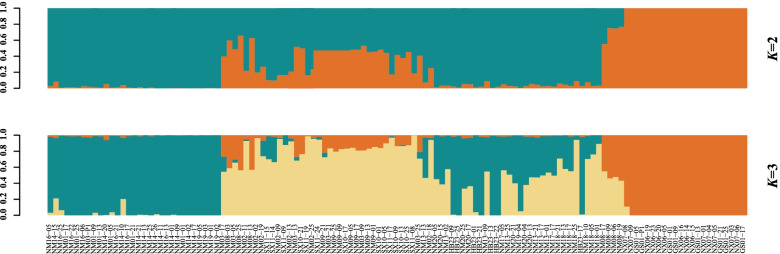
Population structure analysis of the 125 *C. mongholica* accomplished from *K* = 2 to *K* = 3. Note: each inbred line is represented by a thin vertical bar, which is partitioned into two colored segments (orange = group A, green = group B) on the x-axis when *K* = 2, with lengths proportional to the estimated probability on the y-axis

The structure clustering analysis estimated that two genetic groups (i.e., *K* = 2) were most likely, as determined by the Admixture software (Fig. 4 and Additional file 2: Fig. S1). Under *K* = 2, the 18 populations clustered into a small group with four populations (NX06, NX07, NM08 and GS01) and two individuals (NM03-09 and NM03-13) from the NM03, and a large second group with all the remaining populations and individuals (Fig. 4). Similar to the phylogenetic results, individuals from the NM03 population were present in both groups. Under *K* = 3, NX06, NX07 and GS01 populations remained unchanged, while some individuals (NM08-06, NM08-09, and NM08-19) from the NM08 population formed a new cluster with these three populations. In addition, the previous large group branched, now comprising 50 individuals, respectively.

**Fig. 5:**
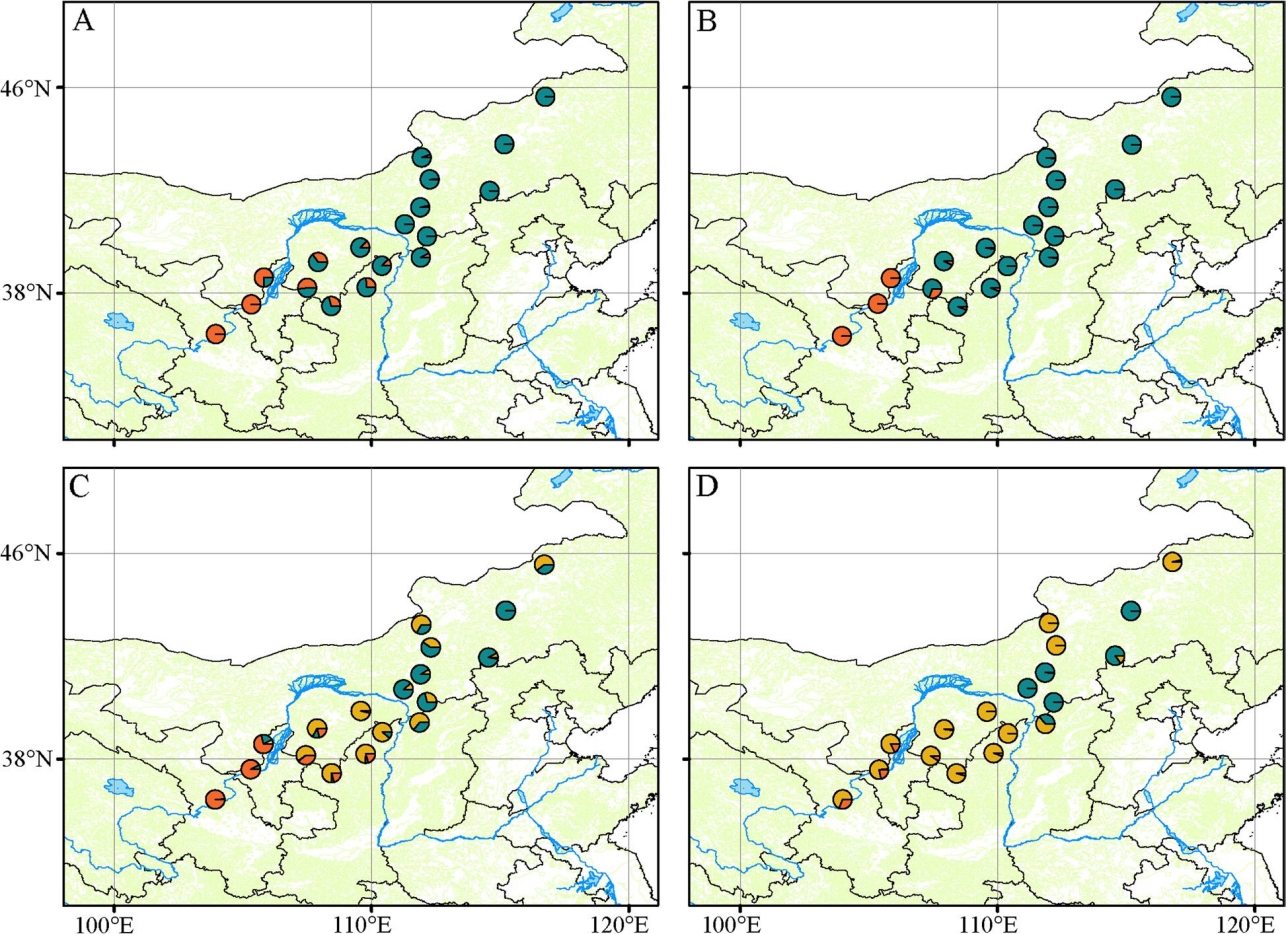
Spatial population structure based on spatial and non-spatial construct models. (A) and (B) Population admixture estimated with *K* = 2 using non-spatial model and spatial model, respectively. (C) and (D) Population admixture estimated with *K* = 3 using non-spatial and spatial model, respectively

conStruct cross-validations (Additional file 2: Fig. S2) showed that the spatial model was marginally superior to non-spatial model continued to improve slightly as subsequent clusters were added up to *K* = 5, indicative of overestimating the number of potential clusters. For the spatial model, the predictive accuracy was highest *K* = 2. Thus, the spatial model at *K* = 2 sufficiently described the population structure, and the clustering patterns of spatial and non-spatial models were very similar (Fig. 5), indicating the contribution of isolation by distance (IBD) to the population structure was small.

The results of ML tree and PCA for the 125 individuals were basically in agreement with that of the structure cluster analysis. As a matter of convenience, we would named the four populations NX06, NX07, NM08 and GS01 as group A (Helan Mountain group), and the remaining 14 populations as group B (Daqing Mountain-Grassland group).

At the species level, the hierarchical analysis of molecular variance (AMOVA) results showed that the majority of the observed genetic variation could be attributed to differences within populations (90.97%) rather than to the variation among populations (9.03%) (Table 2).

**Table 2:** Analysis of molecular variance for among and within populations of the studied *C. mongholica* individuals

Source of variation	*df*	Sum of squares	Variance components	Percentage of variation (%)
Among populations	17	31.461	0.077	9.03
Within populations	232	180.911	0.880	90.97
Total	249	212.372	0.857	100

### Partitioning genomic variation to IBD and IBE

**Fig. 6:**
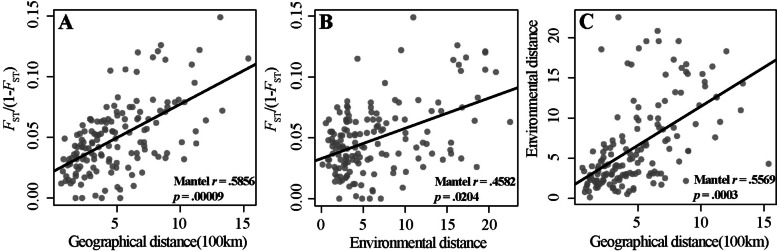
Isolation by distance and environment using Mantel test. (A)Pairwise genetic distance *F*_ST_/(1-*F*_ST_) is significantly associated with environmental distance and (B)geographic distance. (C)Geographic distance is significantly correlated with environmental distance

Gene flow patterns depend on environmental or geographic conditions; therefore, we tested IBD and environment (IBE). A significant correlation between pairwise *F*_ST_/(1-*F*_ST_) and geographic distance (Mantel *r* = 0.5856, *p* = 0.00009; Fig. 6A) was detected by the Mantel test, indicating a significant pattern of IBD. We also identified a significant pattern of IBE(Mantel *r* = 0.4582, *p* = 0.0204; Fig. 6B). The autocorrelation between environmental and geographic distances was also strong (Mantel *r* = 0.5569, *p* = 0.0003; Fig. 6C).

**Fig. 7:**
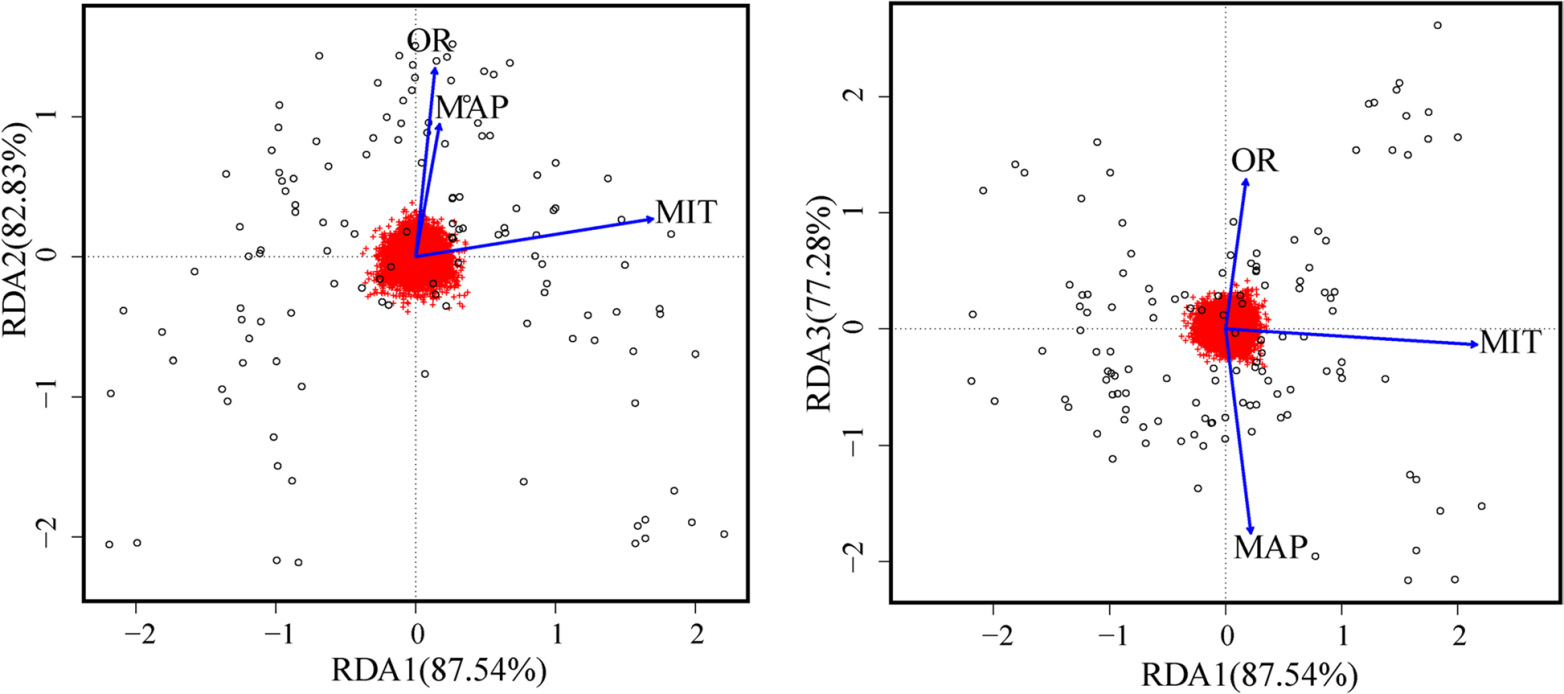
Biplot of redundancy analysis (RDA) of the relationship between genomic variation and three environment variables. The SNPs were in red (in the center of each plot), and the individuals were the black circles. The blue vectors were the environmental predictors

The variance inflation factor (VIF) was less than 10 for all variables included in the RDA. The first axis of RDA accounted for 87.54%. the second axis of RDA accounted for 82.83%, while the third accounted for 77.28% of the environmental variables’ relations (Fig. 7). In addition, MIT showed the greatest number of associated SNPs according to the latent factor mixed models (LFMM) (Additional file 2: Fig. S4).

## Discussion

Recently, genomic resources have allowed us to study genetic mechanism of evolution in more detail. Many researchers have been studying the genetic information of *C. mongholica*, unravelling the molecular basis for protecting its genetic diversity [6, 17]. *C. mongholica* is a plant species with a small genome size (< 400 Mb), for which no complete reference genome exists. Therefore, studies of *C. mongholica* genomics using a genome resequencing-based approach that requires a complete reference genome are limited. SNPs are currently markers of choice for several types of genetic diversity studies in *C. mongholica*. Using the GBS methodology on genomic the DNA of *C. mongholica*, we developed the first genomic-SNP markers for this important drought-tolerant shrub species.

### Genetic diversity

Previous studies have shown that the genetic diversity of the widespread plant species is higher than that of the narrowly distributed [18], and compared to annual and perennial herbaceous plants, perennial woody plants tend to have higher genetic diversity at the species level and within populations [19–21]. As a long-lived perennial shrub plants widely distributed in arid and semi-arid areas, the NM17 population of *C. mongholica* was found to have highest *He* value (0.70), whereas the NM14 had the lowest (0.28) (Table 1)*.* This finding suggests that individuals from NM17 are potential source of useful and rare genes for widening the genetic base of breeding populations derived from *C. mongholica*. Thus, a greater effort should be directed toward collecting specimens from these regions. Compared to coordinal plants, the *He* value of *Salvia yunnanensis* and *Salvia miltiorrhiza* as revealed by Simple Sequence Repeats markers were 0.28 and 0.15, respectively [22], while *C. mongholica* showed slightly higher genetic variation at the species level.

Gene flow plays an important role in the genetic differentiation of population. It increases the genetic variation within populations and a decrease in differentiation among them, which is the opposite effect of the genetic drift [23]. In addition, the genetic differentiation of natural populations is thought to be a dynamic process which relies on an equilibrium relationship between gene flow and selection [23]. In this study, the estimation of gene flow of these populations of *C. mongholica* populations was > 1 (Table 1), indicating it was high enough to resist the genetic differentiation among them caused by genetic drift, thus achieving a homogenizing effect [24].

Our results revealed that higher genetic variations in the *C. mongholica* germplasm were due to differences within the populations and were in line with those of a previous study [7]. Wu characterized the *C. mongholica* in China using ISSR markers and found higher variations within populations (78.98%) than among them (21.02%). Pollen spread and seed dispersal are the most important determinants of gene flow. In nature, *C. mongholica* is mainly pollinated by wind and insects [25], and the long-distance transmission of pollen provides the chances of long-distance pollen transmission opens opportunities for genetic migration among different populations [26]. In the natural communities, *C. mongholica* usually depends on seed propagation, and long-distance seed dispersal must depend on seed propagation, thus, on frugivorous animals [2]. Frugivores have a limited home range and typically disperse only a few seeds [27]. Therefore, we presume that the contribution of seed dispersal to the homogenization of *C. mongholica* populations is likely small and that the frequent gene flow between them might be caused by wind-dispersed pollen or pollinators.

### Population structure

Our assignment test results based on the ML tree separate *C. mongholica* individuals into two groups. This division is consistent with their geographical origins and genetic backgrounds [3]. *C. mongholica* may have evolved from ancestors initially distributed in the Hengduan mountain region of western Sichuan and northwestern Yunnan and gradually expanded to the Loess Plateau and Inner-Mongolia Plateau [3]. The migratory history of *C. mongholica* is complicated, especially when migrating from the Hengduan mountains region in southwest of the Loess Plateau in northwest China, which explains why the genetic relationship of some *C. mongholica* individuals did not well correlate with the geographical origins in this study. For example, “NM02-18” and “NM03-25” were from Dalad Banner Nei Mongol and Otog Banner Nei Mongol, clustered in the same clade and displayed relatively close genetic distance (Fig. 1 and Fig. 2). However, “NM03-13” and “NM03-25” were both from Otog Banner Nei Mongol, but clustered in two different subgroups and displayed relatively distant genetic distances (Fig. 1 and Fig. 2). conStruct gave little support to an overall pattern of IBD in the sampled populations, as also shown by geographically adjacent populations were not always more similar than geographically distant population, such as NM02 and NM08, NM02, and NM20. These results suggest that some populations of *C. mongholica* were isolated in the past, likely as a result of historical events (e.g. geographic isolation or refugia). Geological events [28–30] and subsequent climatic changes during the Pliocene–Pleistocene in northern and western China shaped the distribution and genetic differentiation of species in northern parts of the country [31]. In addition, human activity has also contributed to the fragmentation of *C. mongholica*.

The results of structure was the most likely when *K* = 2, which was supported by the results of ML tree and PCA (Fig. 2 and Fig. 3), confirming the effectiveness of the SNP markers. We used three methods to separate the 18 populations of *C. mongholica* into two groups, the Helan Mountain group (group A) and the Daqing Mountain-Grassland group (group B). Figure 1 clearly shows that NX06, NX07, NM08 and GS01 are located on both sides of the Helan Mountains, and these four populations clusters within the same group of structure analysis. There was a possibility of gene flow among these populations which resulted in genetic similarity and their grouping under the same population. The non-spatial and spatial clustering identified by conStruct was largely congruent with each other also showing a clear east–west transition (Fig. 5). Daqing Mountain-Grassland group clustered a total of 14 *C. mongholica* populations from the northeast side of sampling sites. They were located in relatively wide geographical locations, including grasslands and plains, which may have promoted gene flow among them. In addition, of seven individuals from the NM03 location were used as plant material, two (NM03-09, NM03-13) showed a membership coefficient of more than 50% and clustered in group A, the other five individuals were grouped in the group B (Fig. 4). The intermediate positions of the NM03 population suggest a probability of gene exchange between the two groups. It is speculated that the NM03 may be a transitional population between the two groups.

During the sample collection, we noticed that pink flowers were very rare. Violet-blue color is more favored by insects than pink and has a higher seed-setting rate [25]. However, the population structure indicated that the grouping was not significantly related to the flower colors. Molecular markers reflect the genotype differences in gene expression under specific environmental conditions; thus, they are a product of gene-environment interactions. The phenotype was complex and diverse because of gene regulation and interaction events, which was the main reason why the phenotypic classification and molecular marker classification in many species were partially inconsistent [32–34]. In summary, although identifying *C. mongholica* by color appears intuitive, this trait was susceptible to environmental effects and should be used with caution.

### Environmental adaptation

The testing result of IBD and IBE revealed that geographic and environmental distances were almost equally important to the observed genetic differences implying a certain degree of IBD (Fig. 6). Environment has been widely reported as a strong selective pressure on natural populations [35, 36]. We thus further applied RDA to estimate the impact of environment on genetic variation. Local adaptation studies on climate change contribute to understanding the ability of populations to sustain or adapt to rapid climate change [37]. Adaptive variation is partially drove by environmental factors, which may be mostly driven by temperature, precipitation and minimum temperature for *C. mongholica* [38]. RDA indicated that optical radiation (OR), minimum temperature (MIT) and mean annual precipitation (MAP) were by far the most important variable associated with genetic variation, and the importance of MIT was also confirmed in the LFMM method.

The OR indirectly reflects the temperature, which is a key factor influencing growth and phenology of various species, including *C. mongholica* [8]. OR influences the growth of plants by affecting the metabolic processes such as photosynthesis, respiration, and transpiration, as well as the metabolic processes that affect the synthesis and transportation of organic matter [39]. Additionally, OR can directly affect soil temperature, thus affecting the absorption and transport of water and nutrients. In a previous study of genetic diversity, Jia et al. [14] found that temperature was the most influential factor on genetic variation of *Platycladus orientalis*.

Water availability is commonly recognized as another critical factor delimitating species' distribution in northern China [31, 40]. The precipitation had important implications for the genetic diversity of vascular plants species according to the statistical data of 79 vascular plants [41]. Although MAP is the limitation for plant growth, after long-term adaptive evolution, plants in arid environments can developed different drought-resistant mechanisms (such as resistance genes) as well as genetic structures adapted to the environment [42, 43]. The genetic variation and diversity of the population in arid environments is predicted to increase as a consequence of drought stress, and the rate of evolution of plant populations in these environments is higher than that of the populations in humid environments [43]. On the other hand, the cost of sexual reproduction increased as climatic drought stress increased [44]. For genetic diversity, the effect of aridity should be discussed with caution due to its two aspects.

MIT seemed to be an indispensable factor, which is a limiting factor for the survival [14]. However, the physiological mechanism of *C. mongholica* responding to low temperature is not yet understood. Dissection of this adaptive mechanism should be the objective of future studies.

### Implications for ecological restoration

*C. mongholica* has excellent tolerance to drought and barren soil because of its well-developed roots, and is a frequent pioneer shrub species for sand fixation. It is of great significance to accelerate the greening of northwest China and improve the ecological environment. Owing to its drought resistance, it plays a vital role in forming the landscape of China, especially in the northwestern Loess Plateau [6]. Climate change and human activity cause soil erosion, negatively affecting biodiversity and ecosystem stability. Therefore, protecting and rationally utilizing the wild resources of *C. mongholica* is crucial, as is putting effort into the ecological restoration of its habitats. Genetic resource conservation of wild *C. mongholica* populations should be considered mainly for the ecological restoration of desert areas. In addition to the in situ conservation of the populations with high genetic diversity, ex situ germplasm collection for different purposes, such as breeding and conservation, is also significant for achieving our goals. Furthermore, given that the genetic variation mainly existed within populations, more individuals should be selected and propagated within those used for ecological restoration in these 18 sampling areas. Finally, environmental protection should be advocated in order to increase the local farmers’ awareness of the protection of this valuable species.

The findings of this study provide latest ideas and guidance for the protection, rational development and utilization of *C. mongholica* resources.

## Methods

### Sample collection and DNA isolation

In this study, all of *C. mongholica* shrub materials were originally collected from 18 locations spanning 9º of latitude (~ 36 – 45ºN) and 13º of longitude (~ 103 – 116ºE) across Northwest and North China (Fig. 1 and Additional file 1: Table S1). The sample collection was approved by the Academy of Forestry Science, the Inner Mongolia Autonomous Region, China. Among these materials, the flowers of “NM03-P1” and “GS01-P1” were rare pink in the wild, while those of remaining materials were common violet-blue (Additional file 2: Fig. S3). Each leaf sample was represented by one shrub, spaced at least 25 m apart. The samples were carefully identified by Professor Meng Ji of Academy of Forestry Science based on the descriptions in Flora of China, a voucher specimen was deposited in the Herbarium of Plant Biology Department, Beijing Forestry University with an accession number BJFU-CM117. The leaf materials were dried in silica gel kept in zip-lock bags until DNA extraction. Total genomic DNA was extracted using a Plant Genomic DNA kit (Tiangen, Beijing, China), and its integrity was evaluated on 1% (*w*/*v*) agarose gel. The purity and concentration of DNA in each sample were determined using the NanoPhotometer spectrophotometer (IMPLEN, CA, USA) and Qubit DNA Assay Kit in Qubit 2.0 Flurometer (Life Technologies, CA, USA), respectively.

### Library construction and GBS analysis

GBS libraries were constructed in accordance with the modified protocol [45]. Genomic DNA was incubated at 37℃ with *Msp*I (New England Biolabs, NEB), T4 DNA ligase (NEB), ATP (NEB), and *MspI* Y adapter N-containing barcode. Restriction-ligation reactions were heat-inactivated at 65℃ and digested with the additional restriction enzyme *EcoR*I (NEB) at 37℃. The restricted ligation samples were purified using Agencourt AMPure XP (Beckman). The purified samples were PCR amplified with Phusion Master Mix (NEB) universal primer and index primer to add the index. The PCR products were purified, pooled, and electrophoresed on 2% agarose gel. Fragments between 265 and 315 bps were selected and purified. Pair-end sequencing was performed on the selected tags using the Illumina PE150 platform.

### Processing of Illumina data

Adapter sequences and low-quality bases (base quality < 20) from the tail of each read were removed using Trimmomatic v0.36 [46]. Because the public databases does not contain full genome information for *C. mongholica*, under these circumstances, de novo generation of a GBS reference was constructed following [47]. We selected the sample “NM01-09” with the most tags for stack clustering as the reference sequence. Clean Reads were aligned against the reference sequence using bowtie2 software [48], and then genotyping was performed applying Unified program in the GATK software [49] to predict SNP sites in samples. The predicted results were screened using the SelectVariants program of the GATK software (Key parameters: -restricelesto biallelic-select QD > 10.0). Subsequently, the variant dataset was further filtered using the software vcftools (main parameter -maf 0.01 -minDP 4 -max-missing 0.6).

### Population structure analyses

The genetic structure was characterized using phylogenetic tree construction, PCA, and population structure analysis. The ML phylogeny tree was inferred based on the filtered SNPs using RAxML [50] to determine the evolutionary relationship between populations, and a rapid bootstrapping analysis with 100 bootstraps was conducted. PCA was used to evaluate the structure and calculate eigenvectors and eigenvalues using the software GCTA software [51]. PCA distribution maps were drawn using the R script. Frappe software [52] was used to analyze the group structure, which implements an expectation–maximization algorithm for estimating individual membership in clusters [53]. Briefly, the input file of PLINK—Ped file was created, and the algorithm was used to construct population genetic structure and population lineage information. The number of assumed genetic clusters *K* ranged from 2 to 3. To avoid overestimating the number of potential clusters caused by the presence of IBD, as is often found in continuous populations, we further used conStruct [54] to identify structure in a spatially aware context. conStruct allows for explicit test of discrete versus continuous spatial patterns by estimating the ancestral components of each population. We tested both non-spatial and spatial models. To identify the most favorable fit for the number of clusters(*K*), Admixture software [55] was used to investigate the cross-validation (CV) error, and the minimum CV error, which corresponded to the most favourable *K* value. The genetic structure of each identified population was assessed by calculating nucleotide diversity per base pair (*π*), observed heterozygosity (*Ho*) and expected heterozygosity (*He*) were determined for each population identified. The Stack software [56] was used to calculate *π* and inbreeding coefficient (*F*_*IS*_). Arlequin software [57] was used to calculate the *Ho* and *He*. DnaSP software was used to calculate the pairwise *F*_*ST*_/(1-*F*_ST_) and gene flow (*Nm*) from Hudson et al. [58].

### AMOVA

AMOVA was conducted to quantify genetic variation at two different hierarchical levels: among population within the germplasm group and within populations. Genetic variations were further tested by assigning populations to genetic clusters identified by population structure analyses. The analyses were conducted in Arlequin [57], and the significance levels for the variance components were tested using 1,000 permutation steps.

### Isolation by distance and isolation by environment

To investigate the role of geographic and environmental factors in shaping the spatial genetic differentiation, we calculated:(**A**) the correlation between environmental and geographic distance, (**B**) IBD, and (**C**) IBE. According to the geographic coordinates of the different *C. mongholica* collection sites, the climate data for each sample site were obtained from the WorldCLIM global high-resolution climate database (http://www.worldclim.org/). A total of 17 bioclimatic variables were used to calculate IBE (Additional file 1: Table S3 and S4), including temperature, precipitation, optical radiation and soil pH. The Mantel test was used to assess associations between linearized *F*_ST_/(1-*F*_ST_) and geographic distance and environmental distance with significance determined using 999 permutations in the R package vegan [59].

### Climatic association analysis

To estimate the degree to which genomic variation is influenced by environmental variables, we performed a series of redundancy analyses (RDAs) in the R package vegan [59]. RDA involves a multiple linear regression followed by a PCA on the matrix of regression-fitted values. A dependent matrix of minor allele frequencies for each population and a independent matrice of environmental variables were included. To avoid high collinearity, we excluded those with a VIF over 10 [60]. Finally, we reserved three environmental variables, including OR, MAP and MIT to explain population variation using the RDA function in the vegan package [59]. The other software, LFMM [61], was also used for gene-climate association analysis. As it estimates the hidden impact of population structure, LFMM permits the presence of background levels of population structure (latent factors). The detected SNPs that exhibit an association with the environment were determined according to the z-score. Bonferroni adjustment was used on the z-score values for multiple tests.

## Supplementary Information

**Additional file 1:** **Table S1.** List of 125 *C. mongholica* individuals tested in this study. **Table S2.** Summary of GBS data. **Table S3.** Environmental parameters used in this study, and the mean (±standard deviation) values of 18 locations. **Table S4.** 17 bioclimatic variables in the 18 locations of *C. mongholica*

**Additional file 2: Fig S1.** Plot of ADMIXTURE cross-validation error. **Fig S2.** Cross-validation results comparing the non-spatial and spatial models. **Fig S3.** Pink and viole-blue flowers of *C. mongholica*. **Fig S4.** Manhattan plots representing the distribution of significance values -log10(*p*-value) obtained by the genetic-environment association approach LFMM for 17 bioclimatic variables.

## Data Availability

The plant materials were collected from natural population in geographic distribution of *C. mongholica*. The datasets generated for this study can be found in the NCBI SAR. Bioproject #PRJNA779263. All data generated during the current study are included in this published article and its supplementary information as Additional files 1, 2.

## Abbreviations

*Ho*: Observed heterozygosity
*He*: Expected heterozygosity
*π*: Average nucleotide diversity
PCA: Principal component analysis
RDA: Redundancy analysis
OR: Optical radiation
MIT: Minimum temperature
MAP: Mean annual precipitation
LFMM: Latent factor mixed models
GBS: Genotyping by sequencing
SNP: Single nucleotide polymorphism
MAF: Minor allele frequency
ML: Maximum likelihood
IBD: Isolation by distance
AMOVA: Analysis of molecular variance
IBE: Isolation by environment
VIF: Variance inflation factor
*F*_*IS*_: Inbreeding coefficient
*Nm*: Gene flow
CV: Cross-validation
