# Supplementary material for: Genetic diversity and population structure of Caryopteris mongholica revealed by reduced representation sequencing
Source: BMC Plant Biol. 2022 Jun 17;22:297. doi: 10.1186/s12870-022-03681-y (PMC9205053; doi:10.1186/s12870-022-03681-y)
Supplement: Supplementary file 2 — Additional file 2: Fig S1. Plot of ADMIXTURE cross-validation error. Fig S2. Cross-validation results comparing the non-spatial and spatial models. Fig S3. Pink and viole-blue flowers of C. mongholica. Fig S4. Manhattan plots representing the distribution of significance values -log10(p-value) obtained by the genetic-environment association approach LFMM for 17 bioclimatic variables. [file 12870_2022_3681_MOESM2_ESM.docx]

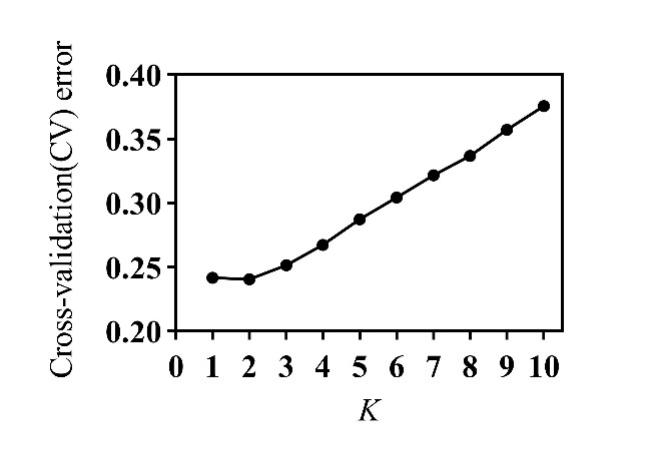


**Fig S1** Plot of ADMIXTURE cross-validation error


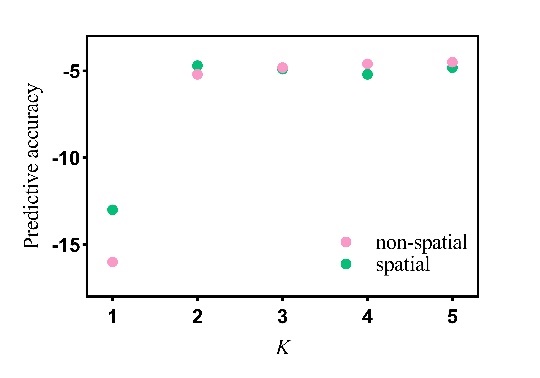


**Fig S2** Cross-validation results comparing the non-spatial and spatial models

**
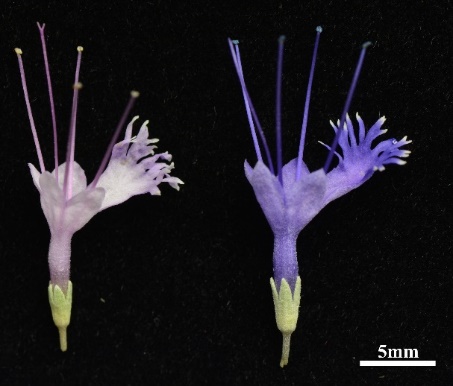
**

**Fig S3** Pink and viole-blue flowers of *C. mongholica*


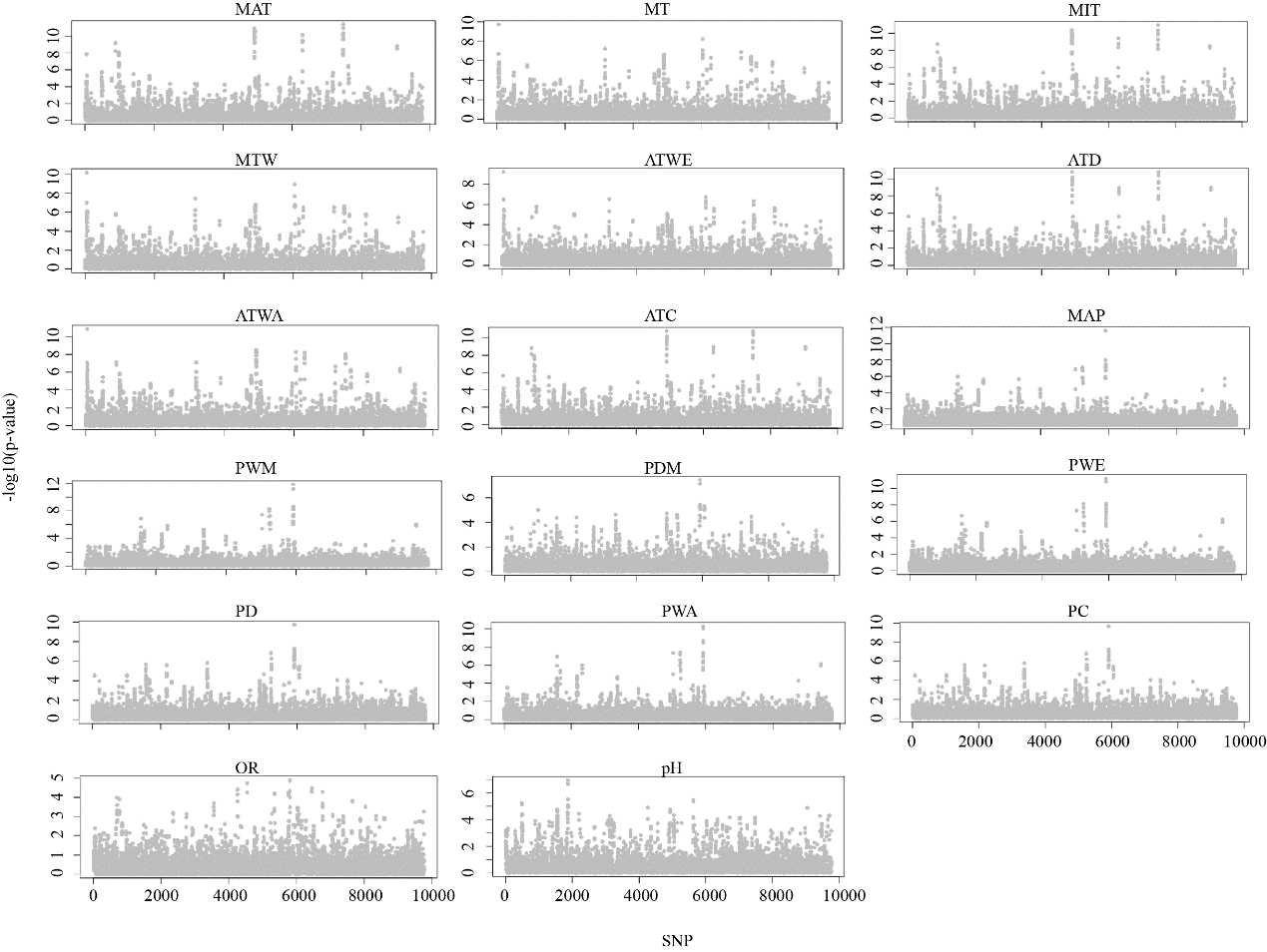


**Fig S4** Manhattan plots representing the distribution of significance values -log10(p-value) obtained by the genetic-environment association approach LFMM for 17 bioclimatic variables.
